# Investigating the sustainability of careers in academic primary care: a UK survey

**DOI:** 10.1186/s12875-014-0205-6

**Published:** 2014-12-14

**Authors:** Raff Calitri, Ann Adams, Helen Atherton, Joanne Reeve, Nathan R Hill

**Affiliations:** Primary Care Research Group, University of Exeter Medical School, Exeter, UK; Division of Mental Health and Wellbeing, Warwick Medical School, University of Warwick, Coventry, UK; Department of Primary Care Health Sciences, University of Oxford, Oxford, UK; Institute of Psychology Health and Society, University of Liverpool, Liverpool, Merseyside UK; Nuffield Department of Primary Care Health Sciences, Radcliffe Observatory Quarter, Woodstock Road, Oxford, OX2 6GG UK

**Keywords:** Sustainability of careers, Barriers to academic primary care careers, Career pathways in academic primary care

## Abstract

**Background:**

The UK National Health Service (NHS) is undergoing institutional reorganisation due to the Health and Social Care Act-2012 with a continued restriction on funding within the NHS and clinically focused academic institutions. The UK Society for Academic Primary Care (SAPC) is examining the sustainability of academic primary care careers within this climate and preliminary qualitative work has highlighted individual and organisational barriers. This study seeks to quantify the current situation for academics within primary care.

**Methods:**

A survey of academic primary care staff was undertaken. Fifty-three academic primary care departments were selected. Members were invited to complete a survey which contained questions about an individual’s career, clarity of career pathways, organisational culture, and general experience of working within the area. Data were analysed descriptively with cross-tabulations between survey responses and career position (early, mid-level, senior), disciplinary background (medical, scientist), and gender. Pearson chi-square test was used to determine likelihood that any observed difference between the sets arose by chance.

**Results:**

Responses were received from 217 people. Career pathways were unclear for the majority of people (64%) and 43% of the workforce felt that the next step in their career was unclear. This was higher in women (52% vs. men 25%; χ^2^(3) = 14.76; p = 0.002) and higher in those in early career (50% vs. senior career, 25%) and mid-career(45%; vs. senior career; χ^2^(6) = 29.19, p < 0.001). The workforce appeared geographically static but unstable with only 50% of people having their contract renewed or extended. The majority of people (59%) have never been promoted by their institution. There were perceptions of gender equality even in the context of females being underrepresented in senior positions (19% vs. males 39%; χ^2^(3) = 8.43, p = 0.015). Despite these findings, the majority of the workforce reported positive organisational and cultural experiences.

**Conclusions:**

Sustainability of a academic primary care career is undermined by unclear pathways and a lack of promotion. If the discipline is to thrive, there is a need to support early and mid-career individuals via greater transparency of career pathways. Despite these findings staff remained positive about their careers.

**Electronic supplementary material:**

The online version of this article (doi:10.1186/s12875-014-0205-6) contains supplementary material, which is available to authorized users.

## Background

Primary care has the broadest remit of all health care areas. It includes care of patients of all ages, socioeconomic status, and geographic origins. Practitioners within primary care are responsible for providing care for a range of acute and chronic conditions, mental health, and social health issues, and the remit of primary care is widening [1]. There is a growing responsibility placed on this sector for the care of patients with more complex and serious health problems [2]. Primary care offers a complex but distinct approach to health care delivery [1,3,4].

Primary care therefore needs a distinct academic discipline working within and alongside health care to support and challenge practice through scholarly activity [5]. Reflecting the complexity of the primary care setting, primary care academics include clinicians, researchers, and teachers from multiple backgrounds who work collaboratively toward the shared goal of improving and delivering quality primary healthcare on the basis of relevant research evidence. Academic primary care is a relatively young discipline but is recognised internationally as making substantive contributions to healthcare [6-8]. A sustainable, multidisciplinary workforce is necessary to ensure that the work performed by academic primary care continues to provide the evidence based provision of primary care.

Workforce sustainability is a challenge within primary care. Academic primary care units across the United Kingdom have undergone considerable change over the past decade [6,9]. UK medical schools are being re-structured into larger, disease or NHS service-focused research groupings with the associated loss of distinctive primary care units in some universities [9]. This means that medical primary care academics (PCAs) and scientist PCA’s who were working as part of a primary care workforce may now be part of a different organisational unit, one that does not necessarily support their professional primary care identity. The Research Excellence Framework has the potential to affect the academic primary care workforce negatively as assessment of research impact operates at the level of the research unit, and not necessarily the departmental level, encouraging specialist research groups [10]. Eight institutions have received additional resources as a result of the formation of the ‘National Institute for Health Research (NIHR) School for Primary Care Research (SPCR)’. However this consolidation of resources may have led to a weakened position of other primary care units outside the NIHR SPCR with uncertainties over wider access of research training opportunities, focused within the school’s member universities [9].

In the clinical context, the complexity and range of NHS services being provided within primary care is increasing [11]. General practitioners have recently received organisational and commissioning authorisation under the government Health and Social Care Act [12]. Medical PCA’s may be balancing clinical and academic responsibilities with increased managerial and administrative workloads. It is possible that medical PCAs may decide to develop their leadership role within the clinical rather than an academic setting, which could result in an increased split between scholarship *in* primary care and scholarship *of* primary care [6]. The loss of medical PCAs, with their clinical and context expertise is potentially a challenge to academic primary care. Further, the Walport report [13] outlined the need for organisational change designed to stimulate and elucidate medical academic pathways while ensuring geographical flexibility in training opportunities, and encouraging equality within medical academic primary care.

At a time of international recognition of the importance of primary care [1], and thus of academic primary care, it is crucial that we understand whether we have the necessary support and structures in place to help PCAs thrive in the current environment, sustaining a workforce. The authors seek to examine the sustainability of academic primary care careers. In preliminary qualitative work by Adams et al. [9] fifteen members of the Society for Academic Primary Care (SAPC) from different career stages and disciplines were interviewed. A number of structural and organisational barriers to sustainability were identified, including the lack of clarity in career pathways and the lack of individual support in career planning. There was an impression of divisions between those from a medical background and scientist PCAs that gave rise to perceptions of disparity in opportunities and available resources. A noted constraint was the need to understand better personal motivations for a career in academic primary care. The research identified a number of organisational factors that might be linked with sustainability. Interviewees commented on the importance of mentorship and a positive organisational culture; and the opportunity for academic freedom and creativity, using discipline-specific knowledge and skills, flexible working arrangements, and a strong shared vision of why their work was important. This work was conducted with a small sample of SAPC members. It is not known whether these issues are applicable to or experienced by the wider academic primary care workforce.

In this study the authors have sought to assess quantitatively the career situation for PCAs in the existing primary care environment, extending the preliminary qualitative work already undertaken.

## Methods

Fifty-three academic departments in the UK conducting research and teaching relevant to academic primary care, were invited to participate, representing the departments in which all current and past SAPC members had worked. The head of each department was invited to complete the survey and asked to cascade the invitation to all staff within their department. The survey was advertised at SAPC’s Annual Scientific Meeting in July 2013. The total survey period was May 2013 to July 2013. The invitation contained information about the purpose and importance of the research and a URL to the online survey. Participants were informed that the survey would take 15–20 minutes to complete. The study was carried out in accordance with the Declaration of Helsinki and ethical approval was given by the University of Warwick Biomedical Research Ethics Committee (REF REGO-2013-118 AM01).

The survey contained questions that captured participant characteristics and aspects of their career, including: current position, whether they possessed a fellowship or tenure, the length of time they had been working in academic primary care, the number of posts they have held, the number of contract changes/renewals, and the number of times they had been promoted. A copy of the full survey is available in Additional file 1. There were questions relating to the clarity of career pathways and organisational culture within academic primary care. The organisational culture questions were divided into sets relating to departmental values (“to what extent does your department/research group value each of the following”: e.g., scholarship, academic freedom and creativity, and multidisciplinary teams, all scored on a 4-point scale: “not at all”, “not much”, “somewhat”, “a great deal”), and departmental demonstrations (“to what extent does your department/research group demonstrate each of the following”: e.g., commitment to staff development, support for staff who need flexible working hours, equality between male and female staff, equality between staff from different backgrounds, all scored on a 5-point scale: “not at all”, “seldom”, “sometimes”, “often”, “consistently”). The 4-point scales were used when asking a participant to rate higher level abstract concepts where a middle option would perhaps not be appropriate and 5-point scales were used to obtain more discriminating answers to questions where the answers are open to validation by the participants. Questions relating to participants’ relationship with SAPC were also asked but were outside the scope of the current research and so are not reported here.

Given the exploratory nature of the study, data were analysed descriptively with the use of cross-tabulation. Career stage (Early career: Lecturer, Teaching Fellow, Assistant Professor, Research Fellow, Research Associate, Academic Clinical Fellow, Research Nurse. Mid-Career: Senior Lecturer, Teaching Fellow, Associate Professor, Senior Research Fellow, Clinical Teaching Fellow, Clinical Community Teacher. Senior career: Professor; Reader, Senior Clinical Tutor), disciplinary background (medical, scientist), age and gender categories were created. For clarity, PCAs are defined as those who have a medical qualification (medical PCAs) and those that do not (scientist PCAs). The Pearson chi-square test was used to determine how likely it was that any observed difference between the sets arose by chance. Missing data were analysed as a function of respondent characteristics to determine whether there was any response bias according to participant characteristics. A P-value of ≤0.05 was deemed statistically significant. Analysis was performed using Stata SE 13.

## Results

The survey had 228 respondents of which 11 did not provide any data - and were excluded - leaving a sample of 217 PCAs (102 medical, 79 scientist, and 33 unknown).

The distribution of the questionnaire invitation in May 2013 - by the 53 heads of department - elicited the majority of responses with 85% of the data coming from this initial contact (n = 193/228). An invitation to participate in the survey was announced at the annual SAPC meeting in July 2013. The remaining responses came following the announcement, but it is unclear whether responses were a direct result of the announcement or simply coincidental.

### Missing data

Logistic regression analyses were conducted on each individual characteristic to determine whether it was uniquely associated with missingness (Table 1). Career stage was associated with missingness. Early career PCAs were more likely to have missing data than senior PCAs (OR = 2.52, 95% CI: 1.22; 5.23). Caution should be taken when interpreting results from this group.

**Table 1:** **Characteristics of participants providing complete vs. partial data**

**Socio-demographic group**		**Providing complete data**	**Missing data**
	**N**	**N (%)** ^**a**^	**N (%)** ^**a**^
**Gender**			
Male	65	48 (74%)	17 (26%)
Female	117	81 (69%)	36 (31%)
Total	182	129 (71%)	53 (29%)
**Age group**			
21-30	26	19 (73%)	7 (27%)
31-40	52	33 (63%)	19 (37%)
41-50	57	40 (70%)	17 (30%)
51-60	34	28 (82%)	6 (18%)
Over 60	12	9 (75%)	3 (25%)
Total	181	129 (71%)	52 (29%)
**Disciplinary background**			
Medicine	102	73 (72%)	29 (28%)
Scientist	79	56 (71%)	23 (29%)
Total	181	129 (71%)	52 (29%)
**Career stage** ^**b**^			
Senior	50	37 (74%)	13 (26%)
Mid	50	30 (60%)	20 (40%)
Early	117	62 (53%)	55 (47%)
Total	217	129 (59%)	88 (41%)

^a^row% may not sum to 100 due to rounding; ^b^Early career = Lecturer/Teaching Fellow/Assistant Prof; Research Fellow; Research Associate; Academic Clinical Fellow, research nurse. Mid-Career = Senior Lecturer/Teaching Fellow/Associate Prof; Principal Research Fellow; Senior Research Fellow; Clinical teaching Fellow, clinical community teacher. Senior career = Professor; Reader, Senior Clinical tutor.

### Sample characteristics

The majority of the workforce (n = 103/181, 57%) were over 40 years of age. Over half of respondents (n = 117/217, 54%) were early career. There were equal numbers (n = 50/217, 23%) of people at mid and senior career. Females outnumbered males (n = 117/182, 64% vs. n = 65/182, 36%) with the majority of people in early career positions being female (n = 66/117, 56% vs. n = 28/65, 43% male). However, of all the females in academic primary care only 19% (22/117) were senior career compared to 38% (25/65) of males, χ^2^(3) = 8.43, p = 0.015. Those from a medical background constituted 56% (n = 102/181) of the sample with no difference between the proportion of medical PCAs and scientist PCA’s in each career stage. Sample characteristics can be seen in Table 2.

**Table 2:** **Socio-demographic characteristics x Career position**

		**Career stage**			
	**N**	**Senior**	**Mid**	**Early**	**Χ** ^**2**^ **(df), p-value**
**Overall Sample** **N (%)** ^**a**^	217	50 (23%)	50 (23%)	117 (54%)	
**Gender** **N (%)** ^**a**^					
Male	65	25 (38%)	12 (18%)	28 (43%)	8.43 (2),
Female	117	22 (19%)	29 (25%)	66 (56%)	0.015
**Total**	**182**	**47 (26%)**	**41 (23%)**	**94 (52%)**	
**Age group** **N (%)** ^**a**^					
21-30	26	0 (0%)	1 (4%)	25 (96%)	
31-40	52	2 (4%)	10 (19%)	40 (77%)	78.68 (8),
41-50	57	17 (30%)	21 (37%)	19 (33%)	0.000
51-60	34	20 (59%)	8 (24%)	6 (18%)	
Over 60	12	8 (67%)	1 (8%)	3 (25%)	
**Total**	**181**	**47 (26%)**	**41 (23%)**	**93 (51%)**	
**Disciplinary background** **N (%)** ^**a,b**^					
Computer Science	1	1 (100%)	0 (0%)	0 (0%)	
Engineering	1	0 (0%)	0 (0%)	1 (100%)	
Medicine	102	28 (27%)	27 (26%)	47 (46%)	
Nursing	5	2 (40%)	2 (40%)	1 (20%)	
Pharmacology	1	0 (0%)	0 (0%)	1 (100%)	21.49 (18),
Physiotherapy	3	1 (33%)	1 (33%)	1 (33%)	0.256
Psychology	16	0 (0%)	2 (13%)	14 (88%)	
Sociology	12	3 (25%)	3 (25%)	6 (50%)	
Statistics	3	2 (67%)	0 (0%)	1 (33%)	
Other	37	9 (24%)	6 (16%)	22 (59%)	
Medicine	102	28 (27%)	27 (26%)	47 (46%)	3.43 (2),
Scientist	79	18 (23%)	14 (23%)	47 (59%)	0.180
**Total**	**181**	**46 (25%)**	**41 (23%)**	**94 (52%)**	

^a^row% may not sum to 100 due to rounding ^b^Given the diversity of other disciplines, disciplinary background was categorised to include those from a medical background vs. any other background.

### Career pathways

When asked if the clarity (or lack of it) of an academic career mattered to them, the majority of people reported that unclear pathways (87%, n = 158/182) and an unclear next career step (80%, n = 148/185) were important issues for them. Academic primary care career pathways (Table 3) were unclear for the majority of people (64%, n = 120/188), but this was reported more frequently by women (72%, n = 84/117vs. men 50%, n = 32/65), χ^2^(3) = 12.95, p = 0.005. Forty three percent of the workforce (n = 80/188) felt that the next step in their career was unclear. This was reported more frequently by woman (52%, n = 61/117vs. men 25%, n = 16/65, ), χ^2^(3) = 14.76; p = 0.002, and those in early career (50%, n = 48/96 vs. senior career, 25%, n = 12/48) and mid-career (45%, n = 20/44; vs. senior career), χ^2^(6) = 29.19, p < 0.001.

**Table 3:** **Career Pathways x Demographic sub-group**

	**Career pathways in academic primary health care are…**				**Χ** ^**2**^ **(df), p-value**
	**Completely unclear N(%)** ^**a**^	**Somewhat unclear N (%)** ^**a**^	**Reasonably clear N (%)** ^**a**^	**Very clear N (%)** ^**a**^	
**Overall sample**	24 (13%)	96 (51%)	67 (36%)	1 (1%)	
**Gender**					
Male	3 (5%)	29 (45%)	32 (49%)	1 (2%)	12.95 (3),
Female	20 (17%)	64 (55%)	33 (28%)	0 (0%)	0.005
**Total**	**23 (13%)**	**93 (51%)**	**65 (36%)**	1 (1%)	
**Career stage**					
Senior	4 (8%)	22 (46%)	21 (44%)	1 (2%)	6.57 (6),
Mid	8 (18%)	21 (48%)	15 (34%)	0 (0%)	0.363
Early	12 (13%)	53 (55%)	31 (32%)	0 (0%)	
**Total**	**24 (13%)**	**96 (51%)**	**67 (36%)**	**1(1%)**	
**Disciplinary background**					
Medicine	11 (11%)	50 (49%)	41 (40%)	0 (0%)	3.77 (3),
Not Medicine	12 (15%)	43 (54%)	23 (29%)	1 (1%)	0.287
**Total**	**23 (13%)**	**93 (51%)**	**64 (35%)**	**1 (1%)**	
	**The next step in your career is…**				**Χ** ^**2**^ **(df), p-value**
	**Completely unclear N (%)** ^**a**^	**Somewhat unclear N (%)** ^**a**^	**Reasonably clear N (%)** ^**a**^	**Very clear N (%)** ^**a**^	
**Overall sample**	24 (13%)	56 (30%)	70 (37%)	38 (20%)	
**Gender**					
Male	3 (5%)	13 (20%)	34 (52%)	15 (23%)	14.76 (3),
Female	20 (17%)	41 (35%)	34 (29%)	22 (19%)	0.002
**Total**	**23 (13%)**	**54 (30%)**	**68 (37%)**	**37 (20%)**	
**Career stage**					
Senior	8 (17%)	4 (8%)	16 (33%)	20 (42%)	
Mid	7 (16%)	13 (30%)	20 (45%)	4 (9%)	29.19 (6),
Early	9 (9%)	39 (41%)	34 (35%)	14 (15%)	0.000
**Total**	**24 (13%)**	**56 (30%)**	**70 (37%)**	**38 (20%)**	
**Disciplinary background**					
Medicine	14 (14%)	28 (27%)	41 (40%)	19 (19%)	1.25 (3),
Not Medicine	9 (11%)	26 (33%)	27 (34%)	17 (22%)	0.741
**Total**	**23 (13%)**	**54 (30%)**	**68 (38%)**	**36 (20%)**	

^a^row% may not sum to 100 due to rounding.

### Academic primary care workforce

The majority of people have worked for only one (68%, n = 148/216) or two universities (20%, n = 43/216) and 50% of all respondents (n = 107/215) have had three or more contract extensions or renewals. The majority of people (55%, n = 119/215) reported not having tenure and most respondents (59%, n = 129/217) have never been promoted in academic primary care by their institution. Forty three percent of people (n = 93/215) have worked in academic primary care for less than 5 years.

### Organisational culture

People perceived their organisational culture to be positive (Table 4). The vast majority of people reported that their academic primary care group valued: scholarship (93%, n = 173/187), academic freedom and creativity (92%, n = 177/192), multidisciplinary teams (84%, n = 164/192), and saw academic primary care as a distinct discipline that enhanced itself through excellence (86%, n = 164/191). Academic primary care groups demonstrated commitment and support to their staff in a number of areas: most people reported that their academic primary care group demonstrated commitment to staff development (67%, n = 128/191), and support for staff who need flexible working arrangements (83%, n = 159/191). However, only around two-thirds of respondents (63%, n = 120/191 ), felt that their departments had a commitment to the retention of good staff. Equality between groups was a key theme, and most (79%, n = 150/191) felt that there was equality between male and female staff. However, approximately half of people reported that there was not equality, or only sometimes equality, between researchers and teachers (49%, n = 91/186), or between different academic disciplines (39%, n = 64/192).

**Table 4:** **Organisational culture**

**To what extent does your department/research centre/group** ***value*** **each of the following?**	**N**	**Not at all** **N (%)** ^**a**^	**Not much** **N (%)** ^**a**^	**Somewhat** **N (%)** ^**a**^	**A great deal** **N (%)** ^**a**^
Scholarship	187	5 (3%)	9 (5%)	73 (39%)	100 (54%)
Academic freedom and creativity	192	2 (1%)	13 (7%)	109 (57%)	68 (35%)
Academic Primary Care as a distinct discipline enhancing Primary Care through academic excellence	191	5 (3%)	22 (12%)	63 (33%)	101 (53%)
Multidisciplinary teams	192	6 (3%)	25 (13%)	76 (40%)	85 (44%)
**To what extent does your department/research centre/group** ***demonstrate*** **each of the following?**		**Not at all or seldom** **N (%)** ^**a**^	**Sometimes** **N (%)** ^**a**^	**Often** **N (%)** ^**a**^	**Consistently** **N(%)** ^**a**^
Commitment to staff development	191	12 (6%)	51 (27%)	81 (42%)	46 (25%)
Commitment to the retention of good staff	191	23 (12%)	48 (25%)	65 (34%)	55 (29%)
Support for staff who need flexible work hours	191	6 (3%)	26 (14%)	93 (49%)	66 (35%)
A ‘rounded’ and not a functional approach to working (i.e. one in which staff are involved in whole projects, and not just in limited components relevant to their skills)	190	12 (6%)	45 (24%)	90 (47%)	43 (23%)
Support for staff in applying for fellowships	189	18 (10%)	32 (17%)	76 (40%)	63 (33%)
Creative, flexible ways of using funds to support individuals	188	17 (9%)	48 (26%)	62 (33%)	61 (32%)
Equity between staff from different academic disciplines	191	23 (12%)	50 (26%)	79 (41%)	39 (20%)
Equity between researchers and teachers	186	31 (17%)	60 (32%)	64 (34%)	31 (17%)
Equity between researchers from different methodological traditions (e.g. qualitative researchers, epidemiologists, clinical trials)	192	13 (7%)	51 (27%)	80 (42%)	48 (25%)
Equity between male and female staff	191	15 (8%)	26 (14%)	63 (33%)	87 (46%)

^a^row% may not sum to 100 due to rounding.

### Mentorship

Previous work [9] by SAPC identified the importance of mentorship. The majority of people (59%, n = 114/194) in academic primary care do not have a mentor (Table 5). Of this group, 69/111 (62%) would like to have one - 67% (n = 47/70) females and 46% (n = 16/35) males. More females reported not having a mentor than males (62%, n = 72/116 females vs. 55%, n = 35/64). The majority of people in early (59%, n = 60/102) and senior (71%, n = 34/48) career positions do not have a mentor. Whereas 34% (n = 11/32) of people in senior career would like one, three quarters (n = 45/60) of those in junior positions would like a mentor.

**Table 5:** **Mentorship**

	**Do you have a mentor? N (%)** ^**a**^			**If no, would you like one? N (%)** ^**a**^		
	**Yes**	**No**	**Total N**	**Yes**	**No**	**Total N**
**Overall sample**	80 (41%)	114 (59%)	194	69 (62%)	42 (38%)	111
**Gender**						
Male	29 (45%)	35 (55%)	64	16 (46%)	19 (54%)	35
Female	44 (38%)	72 (62%)	116	47 (67%)	23 (33%)	70
**Total**	**73 (41%)**	**107 (59%)**	**180**	**63 (60%**	**42 (40%)**	**105**
**Career stage**						
Senior	14 (29%)	34 (71%)	48	11 (34%)	21 (66%)	32
Mid	24 (55%)	20 (45%)	44	13 (68%)	6 (32%)	19
Early	42 (41%)	60 (59%)	102	45 (75%)	15 (25%)	60
**Total**	**80 (41%)**	**114 (59%)**	**194**	**69 (62%)**	**42 (38%)**	**111**
**Disciplinary background**						
Medicine	41 (41%)	59 (59%)	100	36 (62%)	22 (38%)	58
Scientist	31 (39%)	48 (61%)	79	27 (57%)	20 (43%)	47
**Total**	**72 (40%)**	**107 (60%)**	**179**	**63 (60%)**	**42 (40%)**	**105**

^a^row% may not sum to 100 due to rounding.

## Discussion

Career pathways within academic primary care are perceived to be unclear, with staff reporting this as a concern. The unclear pathways have potential significant implications for recruitment and retention in a primary care academic workforce. Of particular concern are the early and mid-career PCAs (the future of the discipline). The results demonstrate that concerns raised within the original qualitative work [9] have wider resonance, and highlight this issue as a top priority in developing a sustainable academic primary care workforce strategy. The findings confirm supporting core values (see Table 4) as a key determinant in career decision-making. These findings suggest a need to focus on offering a clearer vision of a career in academic primary care in order to attract and retain people to the discipline.

The results showed that the academic primary care workforce was geographically static but somewhat unstable in terms of contractual arrangements. The majority of people (88%) had worked in only one or two institutions, but with 41% of people being retained through contract renewals. The majority (59%) have never received a promotion. Despite these threats to career sustainability, people found their organisational culture to be a positive one. Departments’ valued academic scholarship, freedom and creativity, and support commitment to staff development, flexible working arrangements and gender equality. The findings support our qualitative observations that a positive working environment (see Table 4) supports people to stay working within academic primary care. It may be that a work environment that recognises and supports people’s core values ‘compensates’ (in part) for other difficulties related to career pathways. Whilst recognised blocks to career building and advancement need addressing; this should not happen at the cost of continuing to acknowledge and support the vision and values that sustain primary care academics [9].

SAPC has started development of a robust professional development and mentoring programme, currently available to scientist PCAs [14]. The society is now working with the NIHR SPCR and Royal College of General Practitioners (RCGP) to extend the scheme out to medical PCAs. Individuals can be paired with senior, successful academics, a group who reported a clearer career path in the survey. A clearer picture of the route by which people progress in academic primary care, learning from the successes of those who have already managed this, may help early and mid-career people. However, the data do not elicit whether career paths become clearer later on or whether those who stay in academic primary care have more opportunities to shape and define their career paths.

A key finding from the survey was that there appeared to be gender inequalities in career stage, despite the prevailing perceptions of equality. There were fewer females in senior positions than males. It is difficult to know whether the perceived inequality reflects a systemic problem with respect to the promotion of woman to senior positions, as illustrated by schemes such as the Athena Swan charter [15], or an inherent bias within the responders. A further key finding was that the majority of the respondents were over 40 years of age - 57% (n = 103/181). The older age does reflect the population within academic primary care, as it is a ‘hidden’ career in many contexts so a younger workforce may perhaps not necessarily recognise it as an option.

This research has explored the perceptions of individuals’ academic primary care careers in the context of changing culture and structure. The restructuring of academic departments and the likely restriction of resources to those not part of the NIHR SPCR may impact on sustainability. Offering a clearer vision of a career pathway is an important step – showing people what academic primary care is, how to be part of it, and why it matters. But SAPC may need to explore what structures or processes they can implement to help sustain the careers of those who may find themselves in the situation of limited resources and opportunities.

This work is of international significance. A survey of the Australian academic primary care community has demonstrated similar findings [16], reporting a diverse and multidisciplinary workforce that is slightly older than the demographic reported in this UK survey. They expressed concerns about the narrowing of workforce capacity at the mid-career stage. Similar concerns about short-term contracts are highlighted as contributing to problems with retention [17]. Despite these results, they found that people do stay working in academic primary care, perhaps suggesting that the workforce are a self-selecting group for whom contributing to a strategic goal matters. However, further work would be needed to validate this. The Canadian College of Family Physicians also recognise capacity building as a key strategic goal [18] in their blueprint for success.

There are methodological limitations to this work. Critically it was not possible to determine whether the current survey is representative of the academic primary care workforce as the number of staff employed at each of the departments invited to participate was not known. The proportion of PCAs that this paper represents is 29% (n = 217/750) if we use membership of SAPC as the denominator. Although not reported the membership of SAPC has a corresponding percentage of clinical and scientist PCAs to that found in this study. However, this dataset can be considered a reasonable sample of people working within academic primary care and is the largest study in this area to date. It should be noted that this was a convenience sample and may be composed of individuals who have strong – perhaps negative – opinions about the topic matter. Nevertheless, these results highlight the need for a sustained workforce surveillance to understand the changing patterns and pressures within the academic primary care community.

There was significantly more missing data from early career PCAs compared with senior career PCAs. Because we were unable to determine with confidence whether the data were missing at random – that is, we did not know whether the people who did not fully complete the questionnaire would have provided similar responses to the people who did complete the questionnaire items – we could not justify performing multiple imputations on the data. The questionnaire did not provide an opportunity for responders to indicate whether they “don’t know” or whether the question was “not applicable” to them. It was assumed that the higher levels of missingness for early career respondents reflect lack of familiarity with academic primary care, which we were unable to capture owing to flaws in the survey questions. Nevertheless there are data from a number of individuals within each career stage, and the patterns in the data support comments from earlier qualitative work [9], and thus appear sufficient to suggest these issues are experienced by a significant proportion of individuals within academic primary care.

Surveys of the academic primary care workforce [19-21] have previously concluded that research infrastructure was poorly supported by universities and the NHS, compared with the situation in secondary care. In terms of staffing, there was an over-reliance on short-term contract staff (often scientist PCAs), and only a small body of mid-grade staff. Lack of career progression and unclear pathways were similarly flagged as key problems. The situation seems not to have changed (though it is not possible to comment on the actual numbers of staff within each career stage) and is enduring despite the implementation of structures to address training recommended by the Walport report (2005) [13]. The current prevailing context of financial restrictions and organisational restructuring might be influencing the research infrastructure of academic primary care. However, it seems that these issues have been prevalent despite this changing climate.

The results are consistent with earlier qualitative work conducted as part of SAPC’s assessment of academic primary care careers [9]. The earlier work was based on 15 respondents – compared to the 217 in this study - and was a convenience sample. It was not possible to know if the findings were generalisable whereas these results highlight the need for a shared, common vision of what academic primary care is and why it is important. Academic primary care is comprised of individuals from multiple disciplines, and there is a real challenge for SAPC to shape and inform its identity. A clear vision of the discipline may help those joining the workforce to focus their efforts in-line with the common mission.

## Conclusions

Primary care delivers the majority of the work done in the NHS and the primary care workforce is the largest single group in the health service. Academic primary care, the discipline that underpins that work, is relatively small in comparison. Despite the small size, academic primary care continues to make meaningful implementable contributions to improving the quality of primary care practice. Academic primary care makes a direct difference to the care that patients receive and the work that primary care does. Primary care needs academic primary care, and so academic primary care needs a sustainable workforce.

Sustainability of an academic primary care career is undermined by unclear pathways and the lack of promotion. If academic primary care is to thrive, there is a need to support early and mid-career individuals through greater transparency of career pathways. Despite these findings, staff within academic primary care remained positive about their careers.

SAPC is working to address these problems through collaborative work with the RCGP, NIHR SPCR, Clinical Research Networks, the National Institute for Health and Care Excellence, and the wider community to support people in ‘getting in’ and ‘getting on’ in academic primary care. SAPC is attempting to raise the profile of the discipline, address barriers to career progression highlighted here and in previous reports, and support delivery and recognition of the impact from collaborative action. This survey informs that work and highlights the need for continuing investment in monitoring and supporting the academic primary care workforce.

## Additional file

Additional file 1:
**Survey.**

## Abbreviations

NHS: National Health Service
NIHR SPCR: National Institute for Health Research School for Primary Care Research
PCA: Primary Care Academics
SAPC: Society for Academic Primary Care
