# Supplementary material for: Investigating the sustainability of careers in academic primary care: a UK survey
Source: BMC Fam Pract. 2014 Dec 14;15:205. doi: 10.1186/s12875-014-0205-6 (PMC4269923; doi:10.1186/s12875-014-0205-6)
Supplement: Additional file 1: — Survey. [file 12875_2014_205_MOESM1_ESM.pdf]

CONSENT FORM

Title of Research Project: Investigating Careers in Academic Primary Care
Study Number: 148-09-2011
Lead Researcher: Dr Ann Adams

The survey will take 15-20 minutes to complete.

The Executive of the Society for Academic Primary Care (SAPC) is re-examining the sustainability of careers in academic primary care (APC). The motivation for this is a number of significant changes within the context of APC since the last such investigation back in 2003. It is now timely to review the current situation and we would appreciate your help with the research which involves completing their on-line survey.

An earlier, qualitative phase of the study found that lack of clarity about APC career pathways persist, but important factors linked with sustainability were identified at individual and organisational levels. SAPC now wishes to gather information from a UK-wide sample of PC academics, and not just SAPC members, to understand the bigger picture. Findings will be used to inform the SAPC executive's future strategic planning and initiatives aimed at enhancing career sustainability for all PC academics, such as the SAPC mentorship scheme.

Here is the consent form and on entering the organisation code in question 1 you will be able to access the survey on completion of the consent form.

***1. Please insert your university ID number which can be found here http://www.sapc.ac.uk/images/documents/Scoping/University_ID_numbers_scoping.pdf**

[ ]

2. The participant information sheet can be found here http://www.sapc.ac.uk/images/documents/Scoping/PIL_version2.pdf
I confirm that I have read this document for the above study. I have had the opportunity to consider the information, ask questions and have had these answered satisfactorily.

◯ Yes

3. I understand that my participation is voluntary and that I am free to withdraw at any time, without giving any reason.

◯ Yes

4. I agree that, should I withdraw from the study, any information already collected from me with consent may be retained and used within the study.

◯ Yes

5. I understand that relevant sections of any data collected during the study may be looked at by responsible individuals from Warwick University regulatory authorities where it is relevant to my taking part in this research. I give permission for these individuals to have access to these data.

◯ Yes

6. I agree for anonymised direct quotations to be used

◯ Yes

7. I agree that the anonymised written record of my contribution, including quotations, may be kept and used for future research.

◯ Yes

8. I understand that, under the Data Protection Act, I can at any time ask for access to the information I provide and I can also request the destruction of that information if I wish.

◯ Yes

9. I agree to take part in the above study.

◯ Yes

Section 1: Please Tell us about your Career in Academic Primary Care

10. What is your current job title?

- [ ] Professor
- [ ] Reader
- [ ] Senior Lecturer/Teaching Fellow/Associate Professor
- [ ] Lecturer/Teaching Fellow/Assistant Professor
- [ ] Principal Research Fellow
- [ ] Senior Research Fellow
- [ ] Research Fellow
- [ ] Research Associate
- [ ] Academic Clinical Fellow (If yes, please also tick the 'other' box below to tell us about your fellowship. Is it a doctoral, postdoctoral, clinician scientist, professorial or other type of fellowship?)
- [ ] Other (please specify)

11. How is your post funded?

(please tick all the options which apply to you)

- [ ] HEFCE funded
- [ ] Research project funding
- [ ] Personal fellowship (If yes, please state the funder in the 'other' box below, e.g. NIHR, Wellcome, MRC, local funder)
- [ ] NHS funded
- [ ] Other (please specify)

12. Have you ever had a personal fellowship?

- ( ) Yes
- ( ) No (go to question 14)

If yes, please provide details in the box below (Funder, level of award, funding period, amount). Go to question 4

13. What difference has this made to your career?

14. Do you have tenure?

- ◯ Yes (go to question 15)
- ◯ No
- ◯ Unsure

If no, when does your current contract expire?

15. How long have you been working in academic primary care?

- ◯ Less than 1 year
- ◯ 1-2 years
- ◯ 2-5 years
- ◯ 5-10 years
- ◯ 10-15 years
- ◯ 15 years+

16. In how many universities have you worked in APC?

- ◯ 1
- ◯ 2
- ◯ 3
- ◯ 4
- ◯ 5
- ◯ 6+

17. How many different posts have you had in APC?

- ◯ 1
- ◯ 2
- ◯ 3
- ◯ 4
- ◯ 5
- ◯ 6+

18. How many times have you been given a different contract or had your contract renewed or extended in APC? (give an approximate answer if you cannot remember exactly)

- ◯ 0
- ◯ 1
- ◯ 2
- ◯ 3
- ◯ 4
- ◯ 5
- ◯ 6+

19. How many times have you been promoted in APC by your institution?

- ◯ 0
- ◯ 1
- ◯ 2
- ◯ 3
- ◯ 4
- ◯ 5+

Section 2: Please Tell us about Your Work Environment

20. Do you have a mentor?

- ◯ Yes
- ◯ No

If yes, please tell us if this is a formal or informal arrangement, and what difference it makes to you in your career planning and career decisions. (Go to question 22)

21. Would you like a mentor?

- ◯ Yes
- ◯ No

Please use the box below to explain your answer

22. Please tell us about the extent to which your APC department/research centre or group values each of the following:

	Not at all	Not much	Somewhat	A great deal
Scholarship* (see definition below)	◯	◯	◯	◯
Academic freedom and creativity	◯	◯	◯	◯
APC as a distinct discipline enhancing PC through academic excellence	◯	◯	◯	◯
The distinct contributions of different disciplines to APC	◯	◯	◯	◯

Please use the box below to explain your answers. *See 'What is APC?' at http://www.sapc.ac.uk/index.php/sapc-position-statement for a definition of scholarship

23. Please tell us about the extent to which your APC department/research centre or group demonstrates each of the following:

	Not at all	Seldom	Sometimes	Often	Consistently
Commitment to staff development	◯	◯	◯	◯	◯
Commitment to the retention of good staff	◯	◯	◯	◯	◯
Support for staff who need flexible work hours	◯	◯	◯	◯	◯
A 'rounded' and not a functional approach to working (i.e. one in which staff are involved in whole projects, and not just in limited components relevant to their skills)	◯	◯	◯	◯	◯
Support for staff in applying for fellowships	◯	◯	◯	◯	◯
Creative, flexible ways of using funds to support individuals	◯	◯	◯	◯	◯
Equity between staff from different academic disciplines	◯	◯	◯	◯	◯
Equity between researchers and teachers	◯	◯	◯	◯	◯
Equity between researchers from different methodological traditions (e.g. qualitative researchers, epidemiologists, clinical trialists)	◯	◯	◯	◯	◯
Equity between male and female staff	◯	◯	◯	◯	◯

Please use the box below to explain your answers

Section 3: Please tell us your thoughts about Academic Primary Care in Gene...

24. Please tell us your thoughts about career pathways in APC

	Completely unclear	Somewhat unclear	Reasonably clear	Very clear
Career pathways in APC are:	◯	◯	◯	◯

25. Does this matter?

◯ Yes

◯ No

Please use the box below to explain your answer

26. Please tell us about how clear the next step in your own career is to you

	Completely unclear	Somewhat unclear	Reasonably clear	Very clear
My next step is:	◯	◯	◯	◯

27. Does this matter?

◯ Yes

◯ No

Please use the box below to explain your answer

28. To what extent does working in APC......?

	Not at all	Seldom	Sometimes	Often	All the time
Make good use of your discipline-specific knowledge and skills?	○	○	○	○	○
Allow you to address important 'real world' problems?	○	○	○	○	○
Allow you to make a difference to the quality of patient care?	○	○	○	○	○
Allow you to experience job satisfaction?	○	○	○	○	○
Allow you to experience job security?	○	○	○	○	○
Frustrate your personal career ambitions?	○	○	○	○	○

Please use the box below to explain your answers

Section 3: About you and SAPC

29. What is your primary academic discipline?

- ◯ Computer Science
- ◯ Economics
- ◯ Engineering
- ◯ Medicine
- ◯ Nursing
- ◯ Pharmacology
- ◯ Physiotherapy
- ◯ Psychology
- ◯ Sociology
- ◯ Statistics
- ◯ Other (please specify)

30. I am

- ◯ Male
- ◯ Female

31. What is your age?

- ◯ 21-30 years
- ◯ 31-40 years
- ◯ 41-50 years
- ◯ 51-60 years
- ◯ Over 60 years

32. Are you a member of the Society for Academic Primary Care (SAPC)?

- ◯ Yes
- ◯ No

33. If no, have you ever been?

- ◯ Yes
- ◯ No
- ◯ Member, not applicable

Please use the box below to tell us about your membership decisions – about why you are, why you are not, or why you are no longer a member of SAPC

34. Have you ever attended a SAPC conference?

	Never	Once only	More than Once
I have attended a SAPC Annual Scientific Meeting	◯	◯	◯
I have attended a SAPC Regional Conference	◯	◯	◯

35. Are you planning to attend future SAPC conferences?

	Yes	No	Undecided
SAPC Annual Scientific Meetings	◯	◯	◯
SAPC Regional Conferences	◯	◯	◯

Please use the box below to tell us about how useful SAPC conferences are to you

36. Please answer the following questions about SAPC

	Yes	No	Unsure
Are there things which SAPC needs to do differently in future?	○	○	○
Are there things you would like SAPC to do in the future which it does not do now?	○	○	○
Are there things which SAPC does now which you would like it to stop doing in the future?	○	○	○
Are there things which SAPC could be better at?	○	○	○
Are there things which SAPC could do to give it broader appeal to a wider range of academic and clinical colleagues?	○	○	○

Please use the box below to explain your answers

37. A new colleague arrives in your department who hasn't worked in APC before. She asks you, 'So what is APC? What are the core elements of your discipline?' Please use the box below to tell us what you would reply.

38. Is there anything we have not covered which has been an important influence on the sustainability of your career in APC?

- ◯ Yes
- ◯ No

If yes, please use the box below to explain your answer

39. If you would like to share any comments with us about this research or any further thoughts you have about the future direction of SAPC, please use the space below to do so. Thank you.
